# Current Limitations of Intraoperative Ultrasound in Brain Tumor Surgery

**DOI:** 10.3389/fonc.2021.659048

**Published:** 2021-03-22

**Authors:** Andrej Šteňo, Ján Buvala, Veronika Babková, Adrián Kiss, David Toma, Alexander Lysak

**Affiliations:** Department of Neurosurgery, Comenius University, Faculty of Medicine, University Hospital Bratislava, Bratislava, Slovakia

**Keywords:** intraoperative ultrasound, artifacts, pitfalls, training, neurosurgery

## Abstract

While benefits of intraoperative ultrasound (IOUS) have been frequently described, data on IOUS limitations are relatively sparse. Suboptimal ultrasound imaging of some pathologies, various types of ultrasound artifacts, challenging patient positioning during some IOUS-guided surgeries, and absence of an optimal IOUS probe depicting the entire sellar region during transsphenoidal pituitary surgery are some of the most important pitfalls. This review aims to summarize prominent limitations of current IOUS systems, and to present possibilities to reduce them by using ultrasound technology suitable for a specific procedure and by proper scanning techniques. In addition, future trends of IOUS imaging optimization are described in this article.

## Introduction

Standard conventional neuronavigation is a widespread tool for image guidance in brain tumor surgery. It has become standard of practice in many institutions for initial tumor localization, for surgical trajectory planning, and also for assessment of tumor margins during resection (1, 2). However, popularity of various intraoperative imaging methods continues to increase due to the well-known fact, that the accuracy of navigation may become unreliable after brain shift occurs (1, 3–5).

Intraoperative ultrasound (IOUS) has been used during resection of brain tumors for over four decades since early 1980s (6). However, despite the initial enthusiasm, this intraoperative imaging modality was not widely accepted, especially until the end of the millennium. There were various reasons for initial lack of acceptance of IOUS. First, the image quality of older IOUS systems was low. Second, oblique 2D IOUS views were unfamiliar to many neurosurgeons, used to evaluate computer tomography (CT) and magnetic resonance imaging (MRI) brain scans in standard three orthogonal planes—axial, coronal and sagittal. Third, visualization of large lesions in their full extent was problematic due to limited field of view of 2D IOUS probes. Fourth, as standard B-mode ultrasound does not selectively depict the MRI-contrast enhancing portion of diffuse high-grade gliomas, it could not be used for reliable identification and subsequent resection of this most malignant, enhancing glioma tissue. Fifth, many surgeons refused to change their surgical habits and perform horizontal craniotomies only in order to enable sufficient filling of resection cavity with fluid and appropriate ultrasound scanning. Sixth, difficulties in visualizing the bottom of the resection cavity due to IOUS artifacts were repeatedly reported, and this often resulted in insufficient visualization of tumor residua in this area. Seventh, distinct visualization of the entire sellar region during transsphenoidal approach was challenging.

Nowadays, some of these limitations can be minimized using modern IOUS equipment and proper methods of IOUS utilization. However, some pitfalls still persist, and solutions to overcome them are needed.

## Suboptimal Ultrasound Imaging

### Image Quality

The main disadvantage of older two-dimensional (2D) IOUS systems was low image quality (7), mainly due to poor spatial resolution and dynamic range—identification of various brain structures was therefore challenging. Especially imaging of deeper structures such as thalamus and brainstem was insufficient, as low frequency probes of older IOUS systems offered very low spatial resolution. Insufficient imaging quality was evident especially when compared to MRI, which offered superior resolution and tissue differentiation (1).

Comparing to older devices, many new ultrasound systems have significantly better quality—one of the improvements is due to the ability of modern ultrasound systems to electronically and dynamically tune the frequency range of the imaging probe (8). Higher frequency means better image resolution, i.e. a better ability to differentiate two small targets as separate objects (8). However, the drawback of high frequency probes is the reduced penetration of acoustic waves in the tissue due to scattering and absorption (1), and thus insufficient visualization of deeper structures (8). As recommended by Unsgaard et al. (9), to obtain the best image, different probes should be used for imaging of lesions localized in different depth: a 5 MHz (4–8 MHz) probe gives optimal image quality at a distance of 2.5–6 cm from the probe tip, while for superficial lesions a 12 MHz linear probe is ideal as it provides the best image quality for the first few millimeters down to a depth of 4 cm (9). Using different probes for lesions at different depths in a series of 105 IOUS guided-surgeries, Mair et al. (10) introduced a grading system of ultrasonographic visibility for various intracerebral pathologies. Lesions difficult to visualize, having no clear border with normal brain represent Grade 1; lesions clearly identifiable, but with no clear border with normal tissue represent Grade 2; and lesions clearly identifiable, and with clear border represent Grade 3 (Grade 0 was considered for lesions not identifiable by IOUS). Only 8% out of 105 lesions were evaluated as grade 1, and none as grade 0.

Very good IOUS visualization of various non-irradiated brain lesions was repeatedly reported—predominantly of intra-axial tumors like gliomas and metastases (11–14), as well as of extra-axial tumors like meningiomas (11, 15). However, in patients who had received radiotherapy, the quality of ultrasound image often decreases (16). As evaluated by histopathology, a high-end intraoperative ultrasound system was proven to depict glioma (pseudo)borders at least as distinctly as a three-dimensional (3D) T2-weighted MRI image and better than a 3D T1-weighted MRI image (12). When high frequency ultrasound linear probe was used, the accuracy of residual low-grade glioma tissue detection by IOUS imaging was described to be comparable to high-field intraoperative MRI (17).

**Figure 1:**
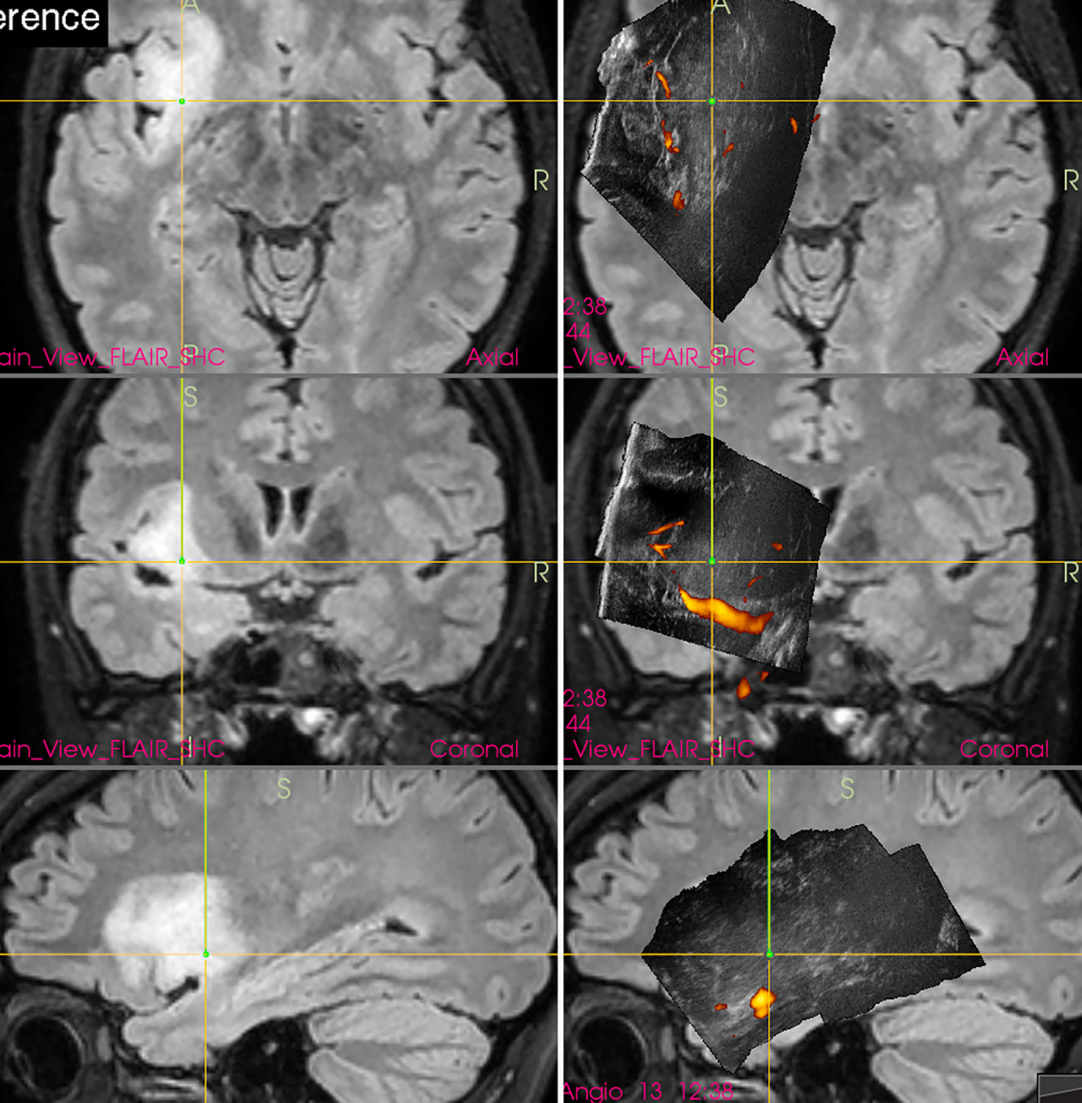
Left-sided insular grade II astrocytoma **(Left column)** preoperative navigation 3D FLAIR MRI sequence (3-Tesla MRI scanner) **(Right column)** pre-resectional 3D IOUS image fused with navigation FLAIR MRI sequence. Note that the tumor tissue is only mildly hyperechoic and less distinctly visualized comparing to MRI. 3D, three-dimensional; IOUS, intraoperative ultrasound; FLAIR, fluid attenuated inversion recovery; MRI, magnetic resonance imaging.

**Figure 2:**
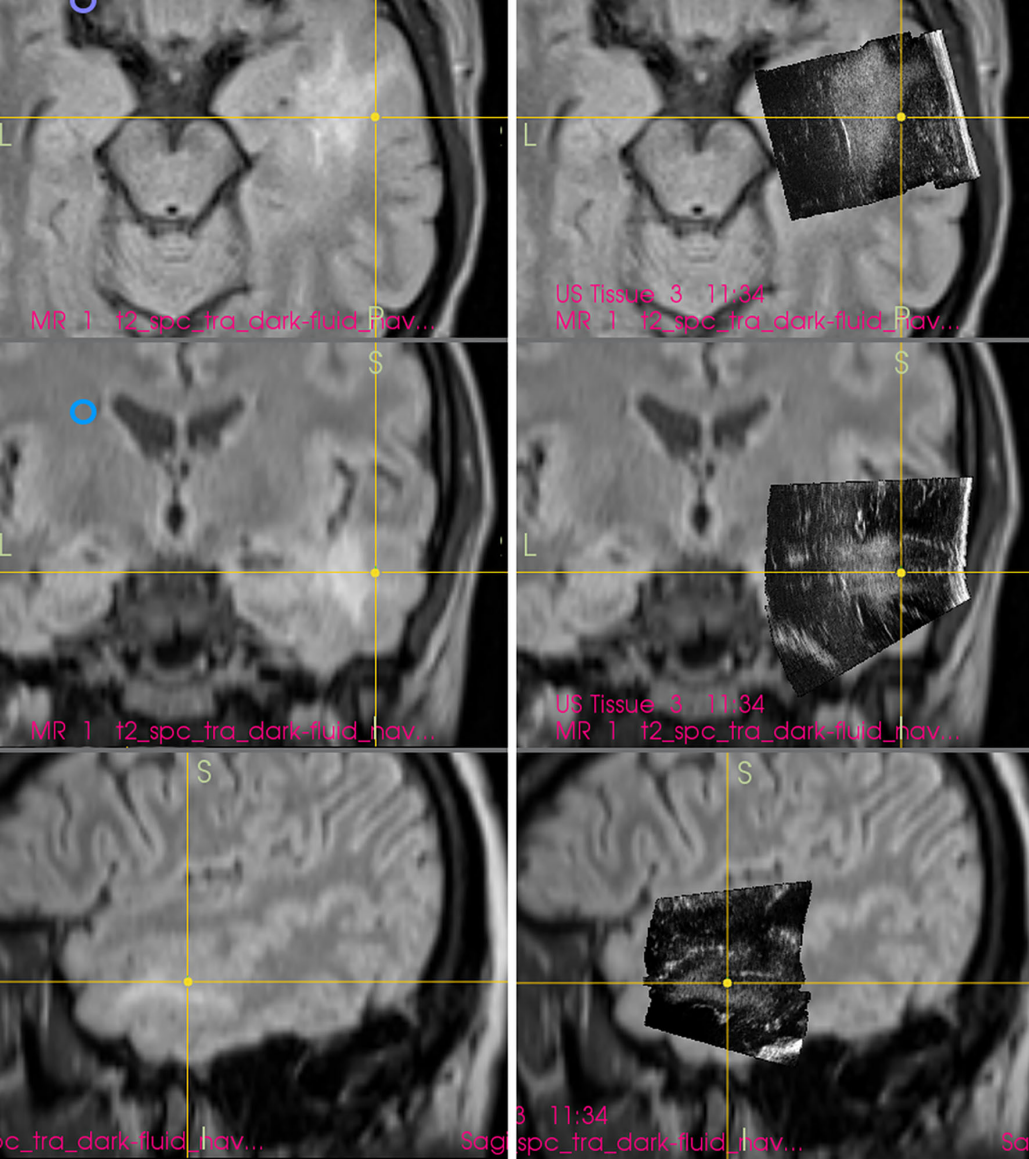
Right-sided temporal grade II astrocytoma **(Left column)** preoperative navigation 3D FLAIR MRI sequence (1.5-Tesla MRI scanner) **(Right column)** pre-resectional 3D IOUS image fused with navigation FLAIR MRI sequence. Note that the hyperechoic tumor tissue is better visualized on IOUS image comparing to MRI. 3D, three-dimensional; FLAIR, fluid attenuated inversion recovery; MRI, magnetic resonance imaging; IOUS, intraoperative ultrasound.

Nevertheless, the newest and perhaps the most detailed data presented by leading Norwegian group showed that MRI is superior in pre-resectional glioma visualization (18) as well as in visualization of small tumor remnants (19). This finding is important (**Figure 1**) despite the fact that in some cases glioma tissue may be better visualized by IOUS, as compared to high-field MRI (**Figure 2**).

**Figure 3:**
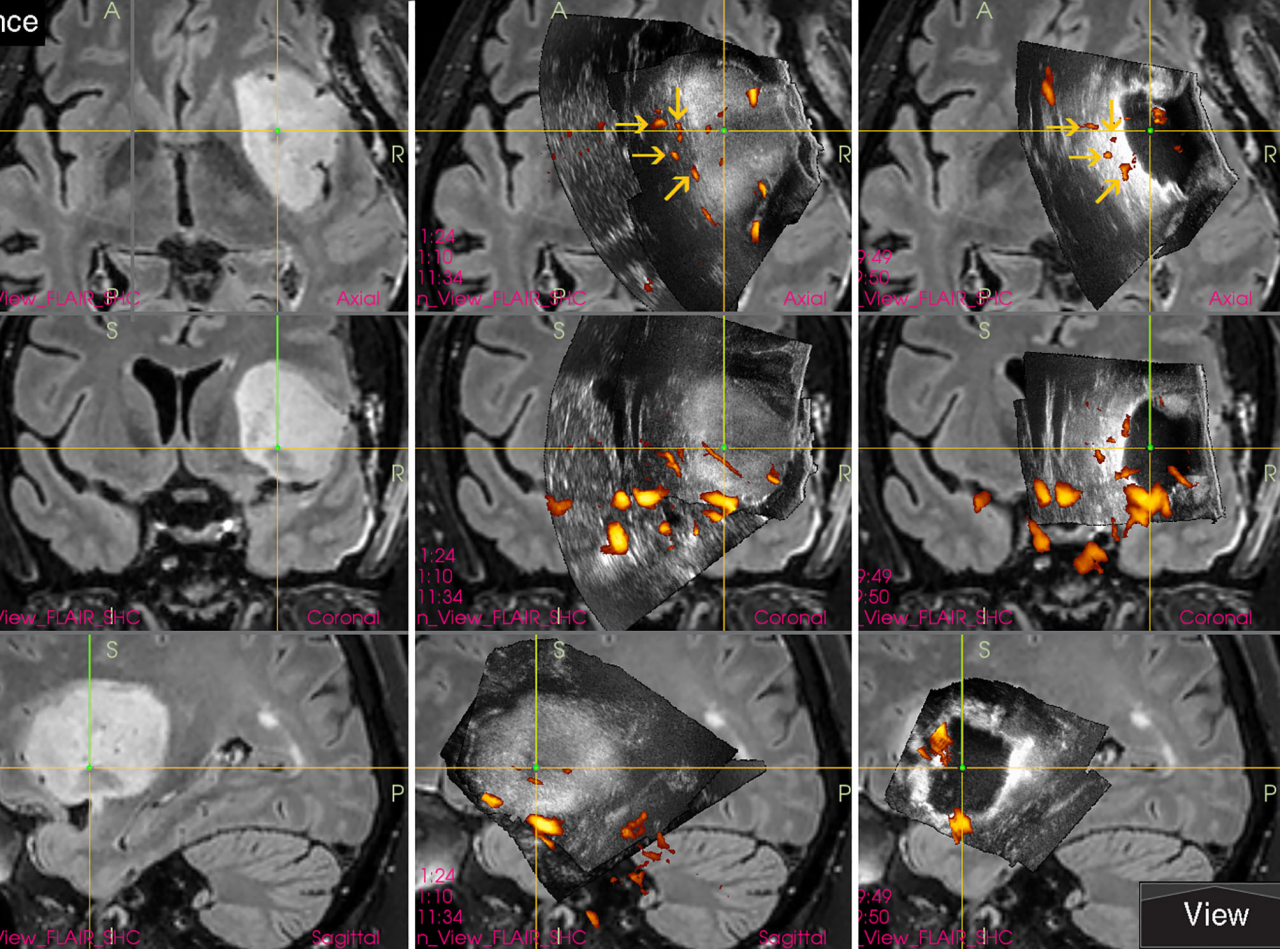
Right-sided insular grade II astrocytoma **(Left column)** preoperative navigation 3D FLAIR MRI sequence **(Middle column)** pre-resectional 3D IOUS image fused with navigation FLAIR MRI sequence. Yellow arrows = lenticulostriate arteries visualized by 3D IOUS power-Doppler mode **(Right column)** 3D IOUS image acquired shortly before the end of resection. Note the close proximity of resection cavity bottom to the lenticulostriate arteries (arrows), intraoperative visualization of perforating arteries helped to prevent iatrogenic injury to them. 3D, three-dimensional; FLAIR, fluid attenuated inversion recovery; MRI, magnetic resonance imaging; IOUS, intraoperative ultrasound.

Interestingly, in spite of reports from prominent neurosurgical centers stating that small deep-seated perforating lenticulostriate arteries (LSAs) cannot be identified by IOUS Doppler imaging (20, 21), these perforators may be in fact depicted (22) by power-Doppler. Using proper methodology and high-end IOUS devices, LSAs may be at least in some patients visualized comparably to MRI (figure). Hence, IOUS power Doppler imaging may serve as an important adjunct during resection of insular gliomas (**Figure 3**) (22). However, prospective studies are needed to evaluate real effectiveness of this relatively new method of intraoperative LSAs identification.

### Anatomical Orientation and Large Lesions Visualization

Most neurosurgeons have extensive experience with the interpretation of CT and MRI images in three orthogonal planes—axial, coronal and sagittal. However, 2D IOUS image is dependent on the orientation of the ultrasound probe, and achieving IOUS scans in at least two exact orthogonal planes may be challenging, especially in small craniotomies (23). Because intraoperative 2D ultrasound views are mostly oblique (24), many neurosurgeons with little or no training/expertise may have considerable orientation problems during 2D IOUS-guided surgeries (25). Understanding the 2D ultrasound image is difficult particularly in areas with no cysts or ventricles visible (24). Another 2D IOUS problem is represented by the fact that ultrasound probes have a limited field of view. It is possible to evaluate only a section of brain tissue during 2D ultrasound scanning, and visualization of large lesions in their whole extent may be problematic.

These pitfalls together with aforementioned suboptimal image quality of older 2D IOUS systems caused preferable use of frameless neuronavigation based on preoperative CT or MRI for brain tumor-surgery guidance by many neurosurgeons (2, 26–31). Unlike 2D IOUS, frameless neuronavigation displays normal and pathological tissue in three orthogonal planes, and also enables preoperative planning of the craniotomy placement and surgical trajectory direction. Only a minority of neurosurgical centers continued in regular 2D IOUS use, mostly because of significant inaccuracy of neuronavigation after the occurrence of brain-shift, considering the fact that 2D IOUS offers a real-time imaging and is unaffected by brain-shift. Others solved the brain-shift problem by using intraoperative MRI for navigation data update (4, 5, 32); this solution however is much more expensive.

**Figure 4:**
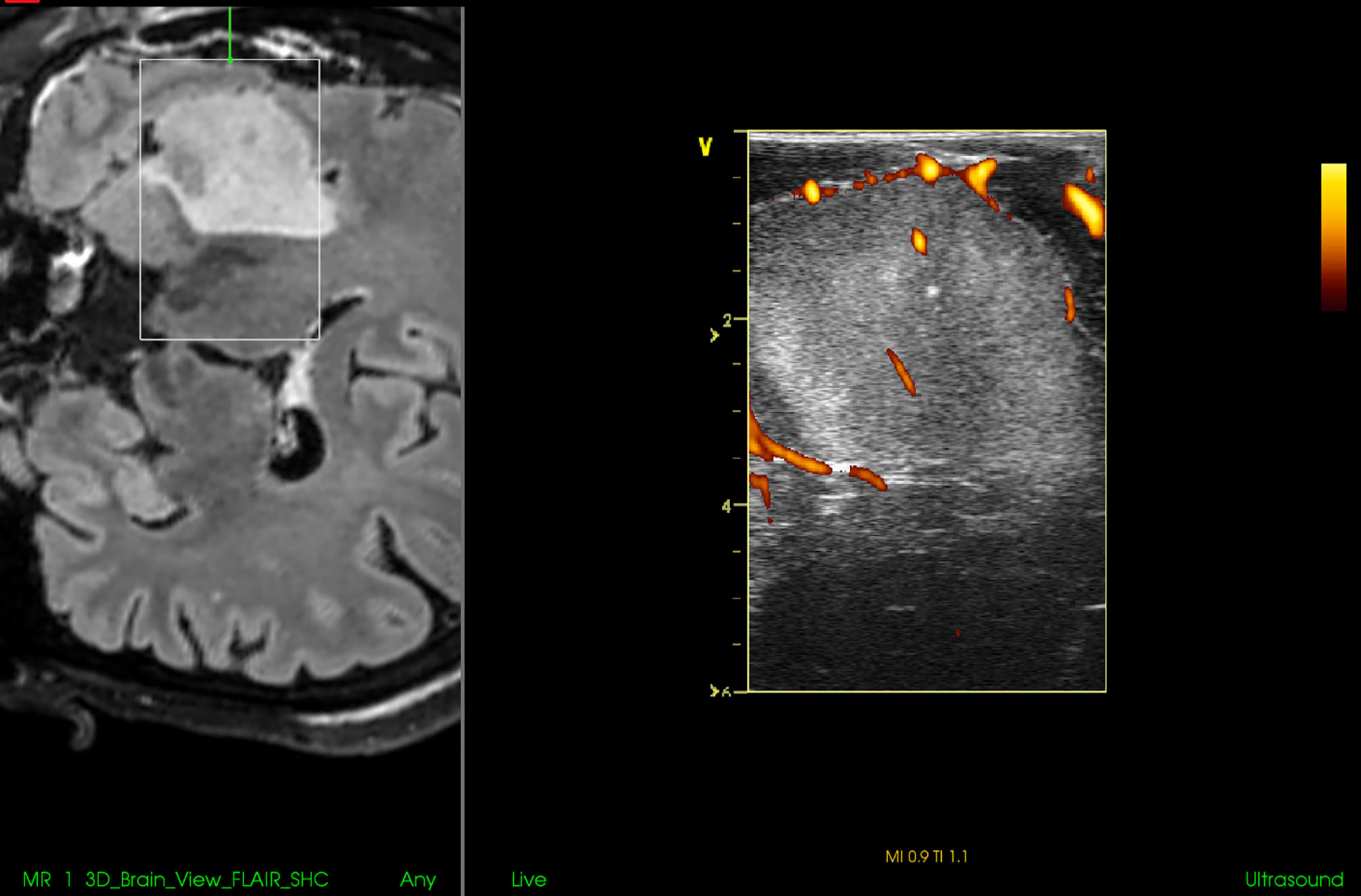
Right-sided insular grade II astrocytoma. Visualization of the tumor before the resection using 12 MHz linear IOUS probe co-registrated with preoperative navigation 3D FLAIR MRI sequence. Note co-registration with navigation MRI facilitates anatomical orientation. 3D, three-dimensional; FLAIR, fluid attenuated inversion recovery; MRI, magnetic resonance imaging; IOUS, intraoperative ultrasound.

In order to simplify the interpretation of ultrasound imagery and allow quantification of brain-shift (33), some groups have connected ultrasound scanner to conventional neuronavigation, digitized the analog video signal from the scanner, and displayed a real-time 2D IOUS image on the navigation computer side by side with the corresponding MRI slice (**Figure 4**) (34–36). However, a much bigger step forward was the integration of neuronavigation and IOUS devices based on a digital interface between the ultrasound scanner and the navigation computer. This type of integration was the basis for the development of navigated 3D IOUS—a system that enables navigation using preoperative 3D MRI or CT data as well as intraoperative 3D ultrasound data (33). Three-dimensional ultrasound data is generated by summation of multiple 2D ultrasound images acquired by moving ultrasound probe freehand over the field of interest; the series of 2D IOUS images are then reconstructed to produce a 3D volume (37). These systems use ultrasound probes equipped with reflective marker reference frames and the position and orientation of the probe during the movement is tracked by means of a navigation camera system. After the scanning, 3D IOUS systems enable surgeons to visualize and navigate the whole volume of normal and pathological tissue that was scanned.

**Figure 5:**
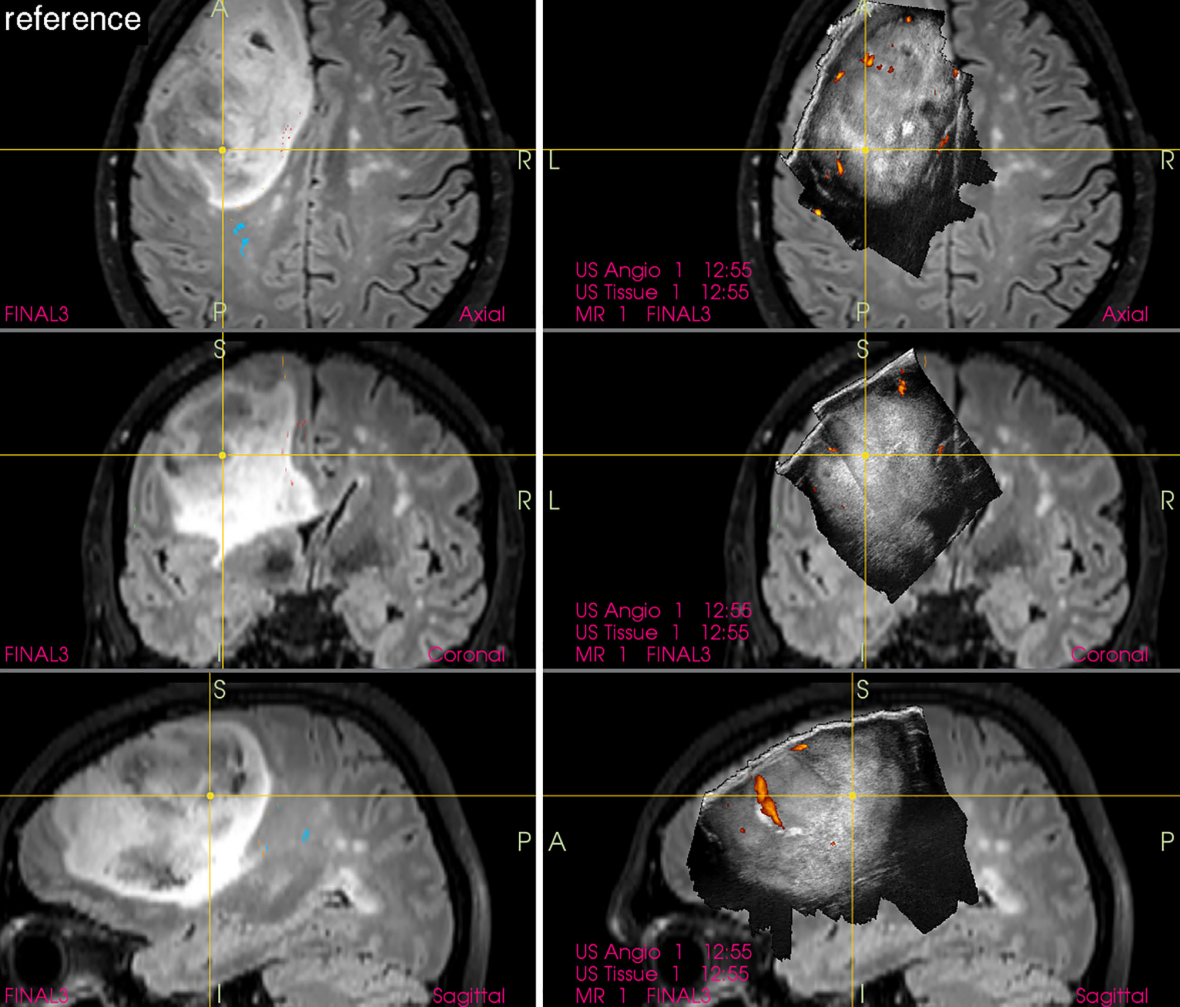
Left-sided frontal grade II astrocytoma **(Left column)** preoperative navigation 3D FLAIR MRI sequence (3-Tesla MRI scanner) **(Right column)** pre-resectional 3D IOUS image fused with navigation FLAIR MRI sequence. Note visualization of the entire tumor on 3D IOUS image despite its large size. 3D, three-dimensional; FLAIR, fluid attenuated inversion recovery; MRI, magnetic resonance imaging; IOUS, intraoperative ultrasound.

By means of combining frameless navigation with ultrasound, the navigated 3D IOUS systems solved prominent drawbacks of stand-alone conventional neuronavigation and 2D IOUS devices—namely the brain-shift problem of navigation as well as the orientation and limited field of view problems of 2D IOUS (25). Even very large lesions, much larger than the ultrasound probe field of view, may be visualized in their whole extent using navigated 3D IOUS systems (**Figure 5**).

Automatic fusion with navigation MRI and/or CT sequence and rendering the ultrasound image in orthogonal planes make the recognition of normal and pathological structures much easier (38). In addition, navigated 3D IOUS provides almost real-time imaging and allows re-scanning of operating field as often as necessary, hence allows effective brain-shift compensation (25). Nevertheless, considering the fact that the “main” part of fused (combined) navigation-MRI/IOUS image is in fact ultrasound visualization of the operating field, knowledge of echogenicity of various normal and pathological brain structures is necessary.

Another benefit of navigated 3D IOUS comparing to standard 2D IOUS is the fact that 3D IOUS is suitable for biopsies of deep-seated supratentorial lesions, as showed by the group of Moiyadi (39).

### Selective Visualization of High-Grade Glioma Portion

**Figure 6:**
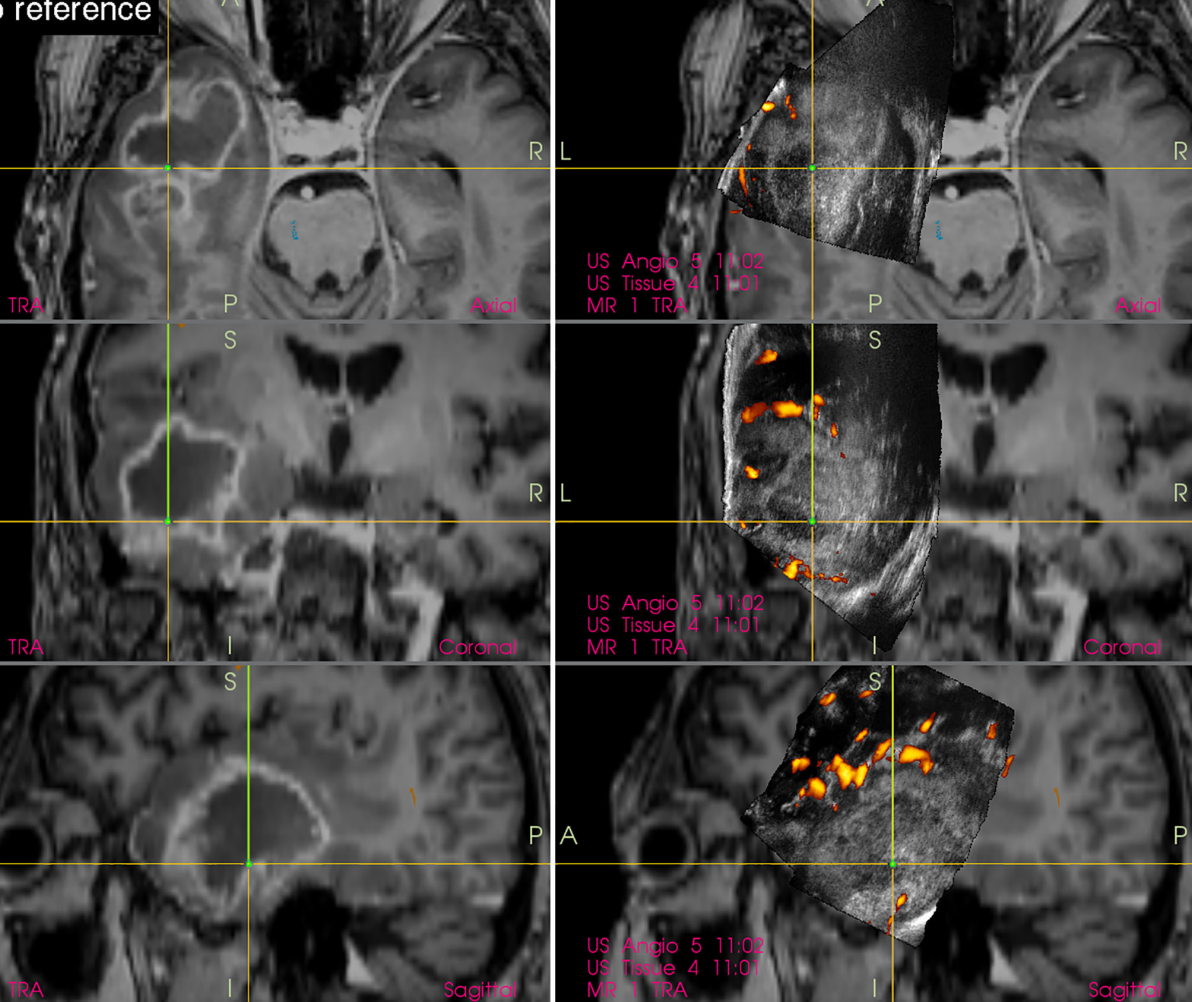
Right-sided temporal glioblastoma **(Left column)** preoperative navigation 3D contrast-enhanced T1-weighted MRI sequence displaying high-grade tumor showing typical ring enhancement **(Right column)** pre-resectional 3D IOUS image fused with navigation MRI. Note that the hyperechoic high-grade tumor tissue is not selectively identifiable on the IOUS image, as the surrounding edematous and infiltrated brain (non-enhancing on MRI) is hyperechoic as well. 3D, three-dimensional; MRI, magnetic resonance imaging; IOUS, intraoperative ultrasound.

Gross-total resection of high-grade gliomas is usually defined as a complete removal of contrast-enhancing glioma tissue evaluated on postoperative contrast-enhanced T1-weighted MRI (40). However, standard 2D or 3D IOUS based on B-mode ultrasound imaging often does not enable reliable selective identification of the most malignant portion of diffuse gliomas (**Figure 6**). Hence, intraoperative evaluation of the extent of resection of high-grade gliomas may be challenging when only B-mode IOUS is used, because both malignant tumor tissue and peritumoral edema, that is in fact usually a mixture of edema and infiltrating tumor cells (41), are hyperechoic.

A potential technique to differentiate between malignant glioma tissue and peritumoral edema is application of ultrasound contrast agents (42). Despite the fact that contrast-enhanced ultrasound (CEUS) agents, which are composed of small gaseous microbubbles, do not penetrate extravascularly (unlike MRI contrast agents which diffuse into the interstitium through disrupted blood–brain barrier), Prada et al. showed that glioblastoma contrast enhancement with CEUS is superimposable on that provided with preoperative gadolinium-enhanced T1-weighted MRI regarding location, margins, morphologic features, and dimensions, with a similar enhancement pattern (42). Hence, CEUS might play a decisive role in the process of maximizing glioblastoma resection (43). Of note however, there is currently no commercially available navigated 3D IOUS system supporting CEUS, all IOUS devices enabling CEUS during brain surgeries are 2D.

**Figure 7:**
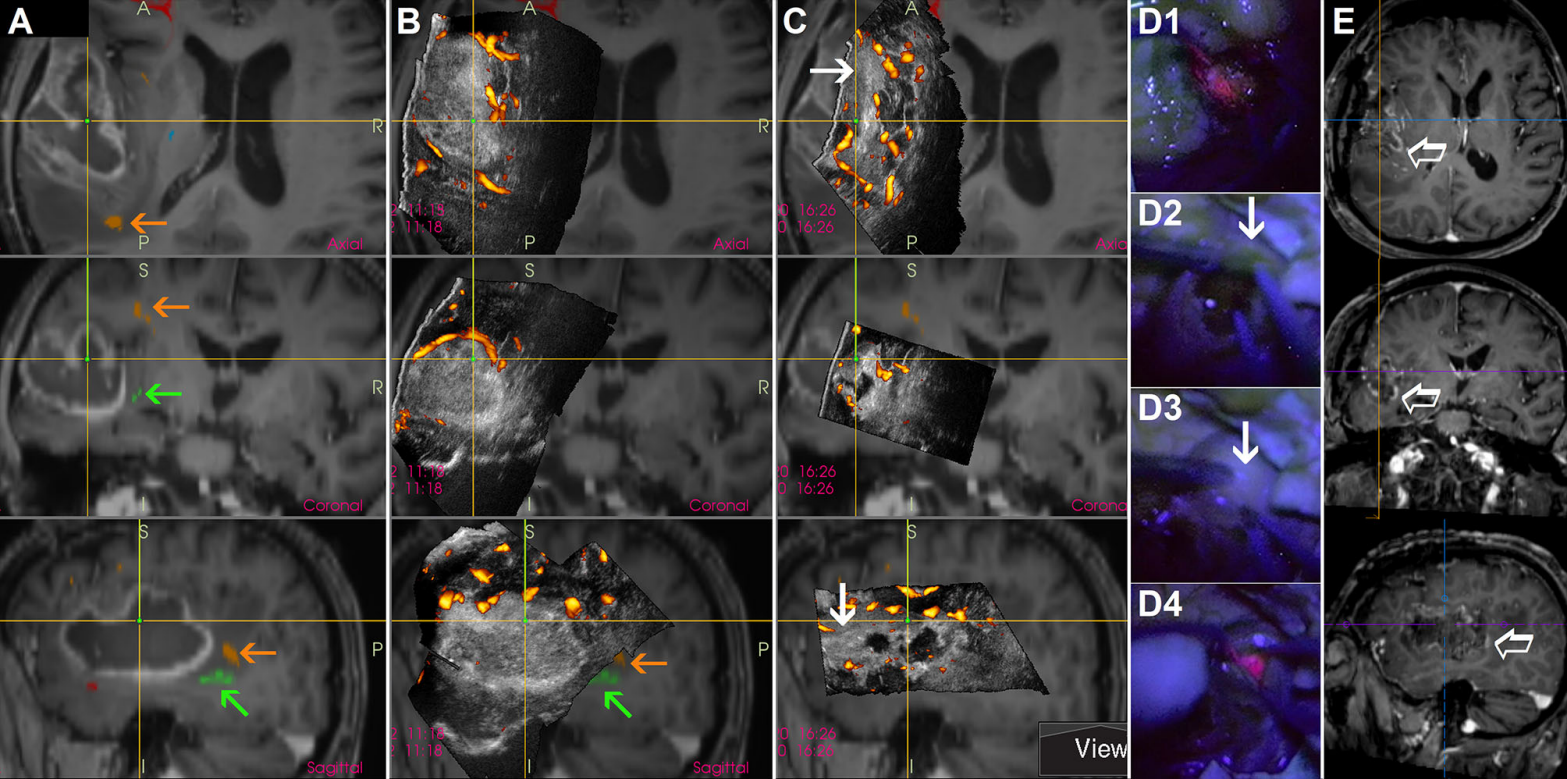
Awake resection of a left-sided temporal glioblastoma guided by direct electrical stimulation, navigated intraoperative 3D IOUS and 5-aminolevulinic acid **(A)** preoperative navigation 3D contrast-enhanced T1-weighted MRI sequence with implemented tractography. Orange arrows = arcuate fascicle. Green arrows = inferior fronto-occipital fascicle **(B)** Pre-resectional 3D IOUS image fused with navigation MRI. Orange arrows = arcuate fascicle. Green arrows = inferior fronto-occipital fascicle **(C)** 3D IOUS image acquired during the resection. Despite the absence of red fluorescence in the anterior part of the resection cavity at the time of IOUS scanning, ultrasound image showed a large nodular tumor residuum in this area (arrows) **(D1)** Distinct red fluorescence observed shortly after the resection beginning **(D2, D3)** Absence of red fluorescence in the anterior part of the resection cavity. Arrow—presumably high grade tumor part identified using actual 3D IOUS scans **(D4)** Distinct red fluorescence after cortical resection **(E)** Postoperative MRI performed 72 hours after the surgery. Empty arrow—contrast-enhancing residual tumor intentionally left in place, electrical stimulation of inferior fronto-occipital fascicle in this area elicited semantic paraphasias. 3D, three-dimensional; MRI, magnetic resonance imaging; IOUS, intraoperative ultrasound.

Another technique to evaluate the extent of high-grade glioma tissue resection is utilization of B-mode IOUS together with 5-aminolevulinic acid (5-ALA) that enables selective malignant tissue visualization (44). In fact, these two methods can be complementary (45), and the combined use of both methods may minimize their pitfalls. Namely, a significant drawback of the intraoperative 5-ALA use is the fact that even a thin layer of intervening low-grade grade tissue is enough to lead to incorrect impression of complete high-grade tumor portion resection (46). Heterogeneous tumors with low-grade parts sometimes cannot be reliably resected by fluorescence-guided surgery alone, in these cases the additional use of intraoperative imaging is required (45, 47). At least in some cases, B-mode IOUS may help to identify larger high-grade glioma residua, and that despite the very challenging differentiation between high-grade tissue and surrounding edema without CEUS (**Figure 7**). In addition, the fact that some high-grade glioma patients may benefit from further resection of T2 abnormality (48, 49) that can be visualized by B-mode ultrasound but not by 5-ALA, underscores the potential benefit of simultaneous use of both methods.

Combination of IOUS and 5-ALA may be potentially useful also when focally malignized low-grade gliomas with no or non-significant contrast-enhancement are resected (50). In such cases, 3D IOUS provides adequate visualization of the whole hyperechoic tumor, while the small foci of anaplasia can be intraoperatively identified by 5-ALA fluorescence using the methodology pioneered by Widhalm et al. (51). This approach helps to achieve an extensive resection of glioma tissue and at the same time helps to identify anaplastic foci in order to avoid a sampling error.

## Ultrasound Artifacts

**Figure 8:**
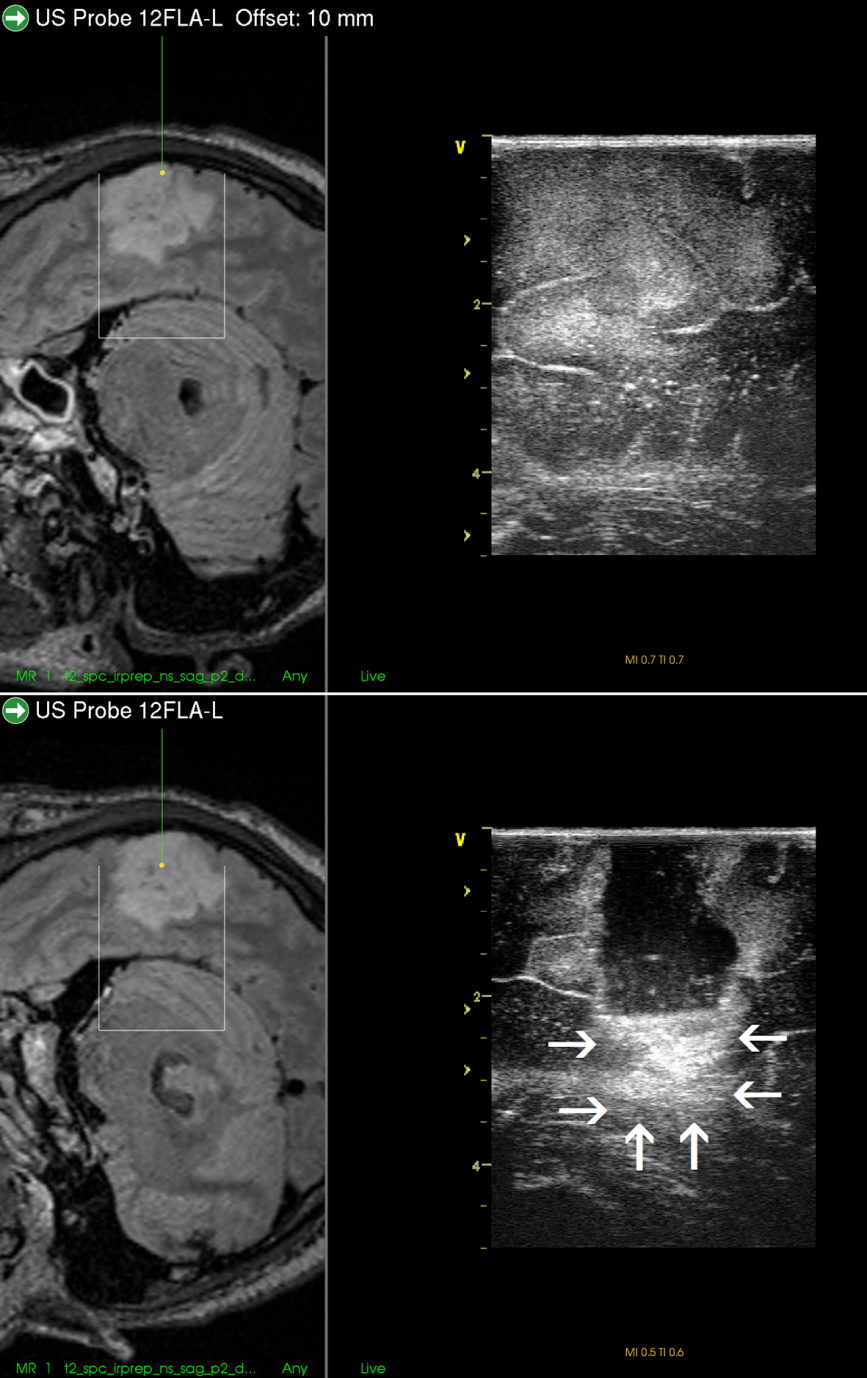
Right-sided temporal grade II oligodendroglioma **(Upper row)** visualization of the tumor before the resection using 12 MHz linear probe co-registrated with preoperative navigation 3D FLAIR MRI sequence **(Lower row)** visualization of the tumor after resection of the central tumor part. Note clear visualization of the tumor residua on the sides of the resection cavity, and large acoustic enhancement artifact at the resection cavity-bottom precluding identification of potential tumor remnant in this area. 3D, three-dimensional; FLAIR, fluid attenuated inversion recovery; MRI, magnetic resonance imaging.

Perhaps the most important pitfall of all neurosurgical ultrasound devices is various ultrasound artifacts (52–54). From practical point of view, the most prominent problem is the acoustic enhancement artifacts (AEAs). These artifacts appear at the bottom of the resection cavity after some tumor debulking (13, 55), when ultrasound probe is placed at the level of brain surface and ultrasound waves penetrate through a higher column of saline solution. The appearance of AEAs is due to a large difference between a very low attenuation of acoustic waves in saline solution and high attenuation of acoustic waves in (normal or pathological) tissue (55, 56). Because AEAs are, similarly as the majority of brain tumors, hyperechoic, the ultrasonic depiction of medial tumor borders after some tumor debulking may be challenging (53). Acoustic enhancement artifacts are especially significant during resections of voluminous tumors and large resection cavities, as the degree of enhancement depends on the distance that ultrasound waves have traveled in saline. Importantly, AEAs may often preclude the detection of tumor remnants at the bottom of the resection cavity and make IOUS unreliable (**Figure 8**) (52). Understandably, this happens mostly towards the end of resection, when brain-shift usually occurs and intraoperative imaging is needed most (53).

Several methods that enable differentiation between AEAs and tumor remnants and estimate the extent of resection were presented: The first possibility is to evaluate the bottom of the resection cavity by moving the probe. In real-time 2D IOUS, the location of the AEAs in the image will move when the position and angle of ultrasound probe has changed (55).

Another possibility to indirectly distinguish AEAs and residual tumor is a comparison between pre-resectional and updated ultrasound image, performed during or after resection. If the hyperechoic area is localized in a region where no tumor was present before the resection, it is most probably a bright artifact and not a real tumor remnant (55, 57).

**Figure 9:**
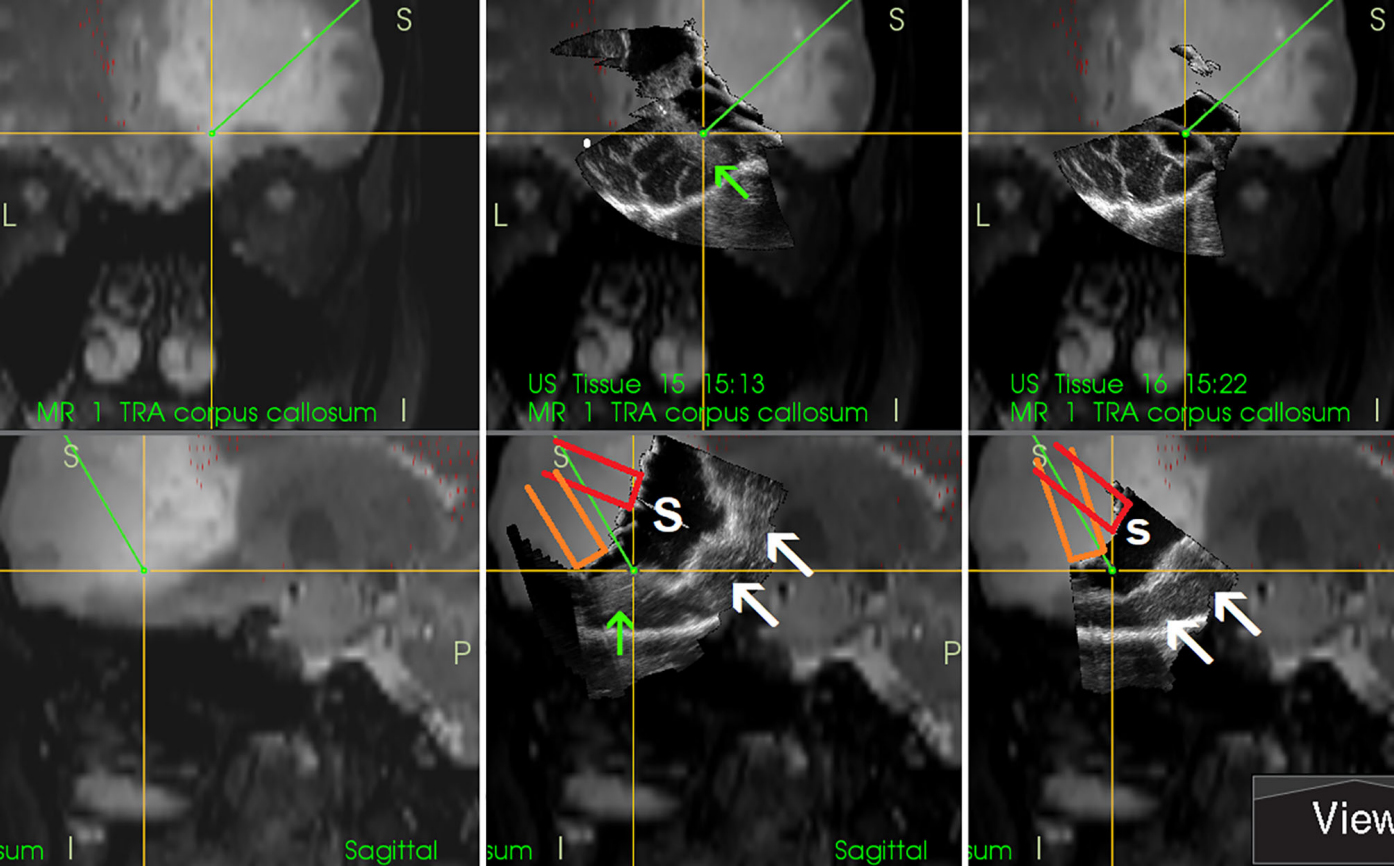
Right-sided frontal grade II oligo-astrocytoma **(Left column)**—3D T2-weighted navigation sequence **(Middle column)** 3D IOUS image fused with navigation MRI. The tip of the pointer (green line) points at small tumor residuum (green arrow) visualized by miniprobe inserted into the resection cavity. Red and orange lines: schematic depiction of the miniprobe position within the resection cavity during the scanning. Note that acoustic enhancement artifacts appeared when the distance between the probe-tip and the scanned tissue became larger (arrows) **(Right column)** The same intraoperative situation as shown in the middle column, 3D IOUS image was acquired after resection of identified tumor residuum. 3D, three-dimensional; MRI, magnetic resonance imaging; IOUS, intraoperative ultrasound; S, hypoechoic saline solution within the resection cavity.

Thirdly, AEAs may be minimized by inserting a small ultrasound probe into the resection cavity (13, 55, 57, 58). By doing so, the column of saline solution between the tip of the miniature probe and scanned tissue at the bottom of resection cavity is smaller than when scanning with a larger probe placed at the level of the brain surface. Shortening the column of saline solution reduces the AEAs at the bottom of resection cavity, and the structures in the medial part of resection cavity can be distinctly depicted (**Figure 9**). However, this method is not without limitations. Small probes have a very limited field of view (43), which may be a significant limiting factor predominantly when these probes are used with 2D IOUS systems (59). Under such circumstances the anatomical orientation may be difficult (43). On the other hand, when used with navigated 3D IUOS, this pitfall may be at least partially minimized, as described by our group (59). Nevertheless, artifacts reduction using mini-probes is certainly not ideal. While frequently allowing depiction of tumor remnants, it is sometimes problematic to maintain the same distance between the tip of the mini-probe and the resection cavity bottom; when this distance becomes larger AEAs appear (**Figure 9**). Next, while some linear probes used for intracavitary visualization allow high resolution imaging (17), image quality of other mini-probes is far from ideal. In addition, even small probes may be too bulky to be safely inserted into the resection cavity in between deliberated bridging veins, which may hinder mini-probe insertion (60).

A new solution for this longstanding problem may be minimizing the AEAs by utilizing the artifact reducing acoustic coupling fluid. This fluid was developed by the group of G. Unsgaard (55, 56); because the fluid attenuates ultrasound energy similarly to normal brain tissue, the AEAs are minimized. Promising results of phase one clinical study were recently published (61).

## Challenging Patient Positioning

**Figure 10:**
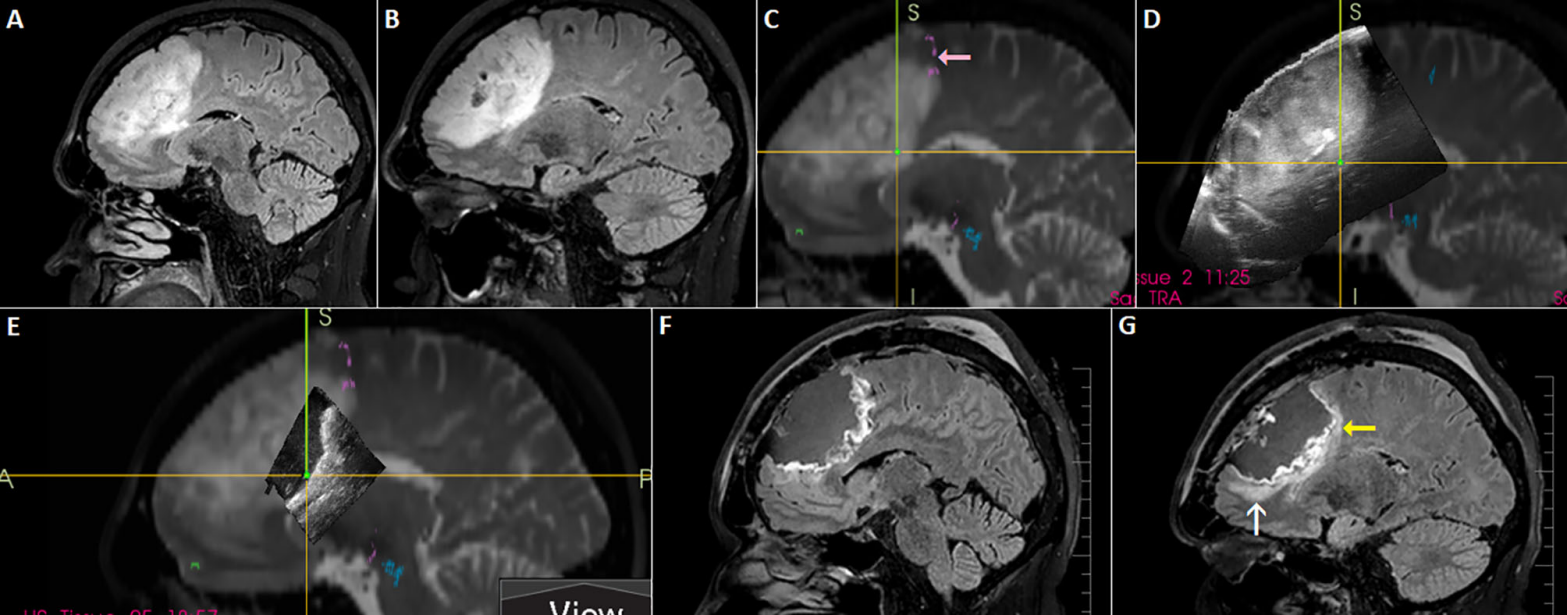
Left-sided frontal oligodendroglioma resected in a semi-sitting position. **(A, B)** Preoperative sagittal FLAIR MRI sequence **(C)** 3D T2 sequence fused with tractography. Note involvement of tracts originating in pre-supplemetary motor area (pink arrow) **(D)** 3D IOUS image fused with navigation MRI **(E)** Incomplete filling of the resection cavity with saline due to non-horizontal placement of the craniotomy resulted in insufficient scanning of anterior part of the resection cavity **(F, G)** Postoperative sagittal FLAIR MRI sequence showing resectable residuum (white arrow), as well as residual tumor involving eloquent tracts (yellow arrow). 3D, three-dimensional; FLAIR, fluid attenuated inversion recovery; MRI, magnetic resonance imaging; IOUS, intraoperative ultrasound.

**Figure 11:**
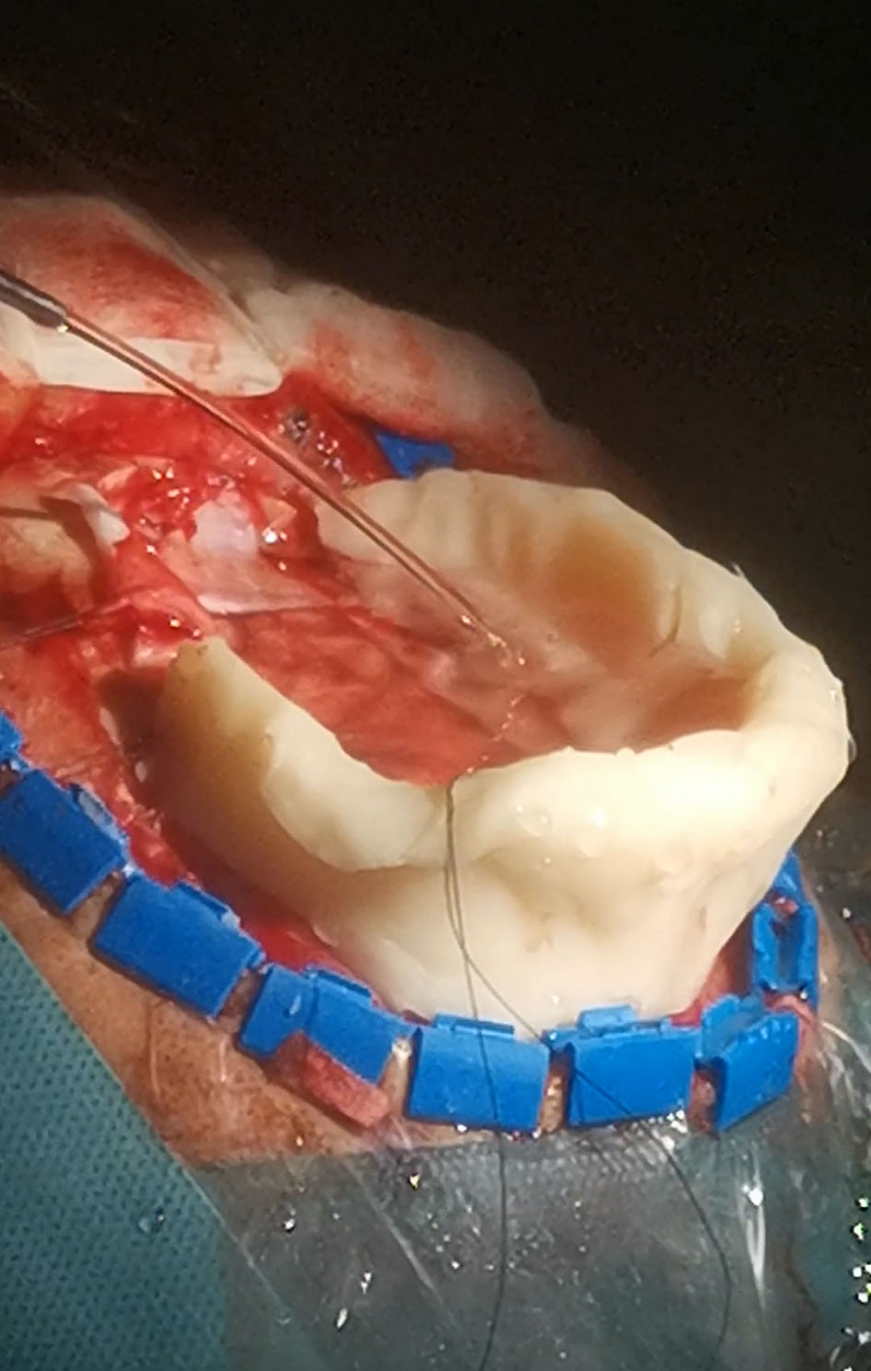
Bone-wax mini-barrier used during removal of a precentral tumor performed in semi-sitting position.

During scanning the operating field with the ultrasound probe placed at the level of brain surface the resection cavity has to be filled with fluid, most often saline solution (9). If air becomes trapped in the resection cavity, the adequate visualization is compromised. At the interface with structures characterized by very low acoustic impedance (such as entrapped air), the sound will be completely reflected and cannot propagate beyond these interfaces—an “acoustic vacuum” will be created (62). Therefore, the position of the patient’s head should enable horizontal position of the craniotomy; in that way fluid will fill a whole resection cavity. However, horizontal placement of the craniotomy may not be optimal in every type of surgery—for example in awake resections of tumors growing close to the Rolandic area and/or supplementary motor area performed in semi-sitting position (which is most comfortable for patients) (**Figure 10**). In order to keep the fluid within the resection cavity in cases when the craniotomy is not placed horizontally, a miniature barrier made from bone-vax may be effectively used (50) (**Figure 11**). This “miniature dam” allows sufficient filing of the resection cavity with fluid and appropriate scanning. Another possible solution in cases with non-horizontal placement of the craniotomy is insertion of the hockey stick-shaped ultrasound probe into the resection cavity, as described by Coburger et al. (17).

## Ultrasound Visualization of Sellar Region

Despite the fact that a variety of IOUS transducers have been applied to transsphenoidal surgery, the significance of endonasal IOUS in the context of transsphenoidal tumor surgeries is still unclear (1). While several reports describe IOUS as a useful adjunct during transsphenoidal resections of microadenomas (63–65) as well of macroadenomas (66–72), IOUS imaging is generally not considered to be a comparable alternative to intraoperative MRI during resections of sellar tumors (73). There are several pitfalls of IOUS use during trassphenoidal procedures, especially during resections of macroadenomas and giant pituitary adenomas. Firstly, while preoperative imaging of these lesions usually includes coronal T1- and T2-weighted and sagittal T1-weighted MRI scans, as well as coronal and sagittal postcontrast T1-weighted MRI scans (74), only very few reports describe ultrasound imaging both in sagittal and coronal planes (66, 67). On the contrary, numerous IOUS probes used for intraoperative imaging during transsphenoidal procedures offer nonintuitive imaging planes most neurosurgeons are unfamiliar with (69). Secondly, imaging results and interpretation are highly dependent on the skills of the investigator and the resolution of many transducers is low (73). Thirdly—there is currently no commercially available ultrasound device that would enable 3D IOUS reconstructions and image rendering in orthogonal planes, which might improve the surgeon’s ability to understand ultrasound imaging of the sellar region (1).

**Figure 12:**
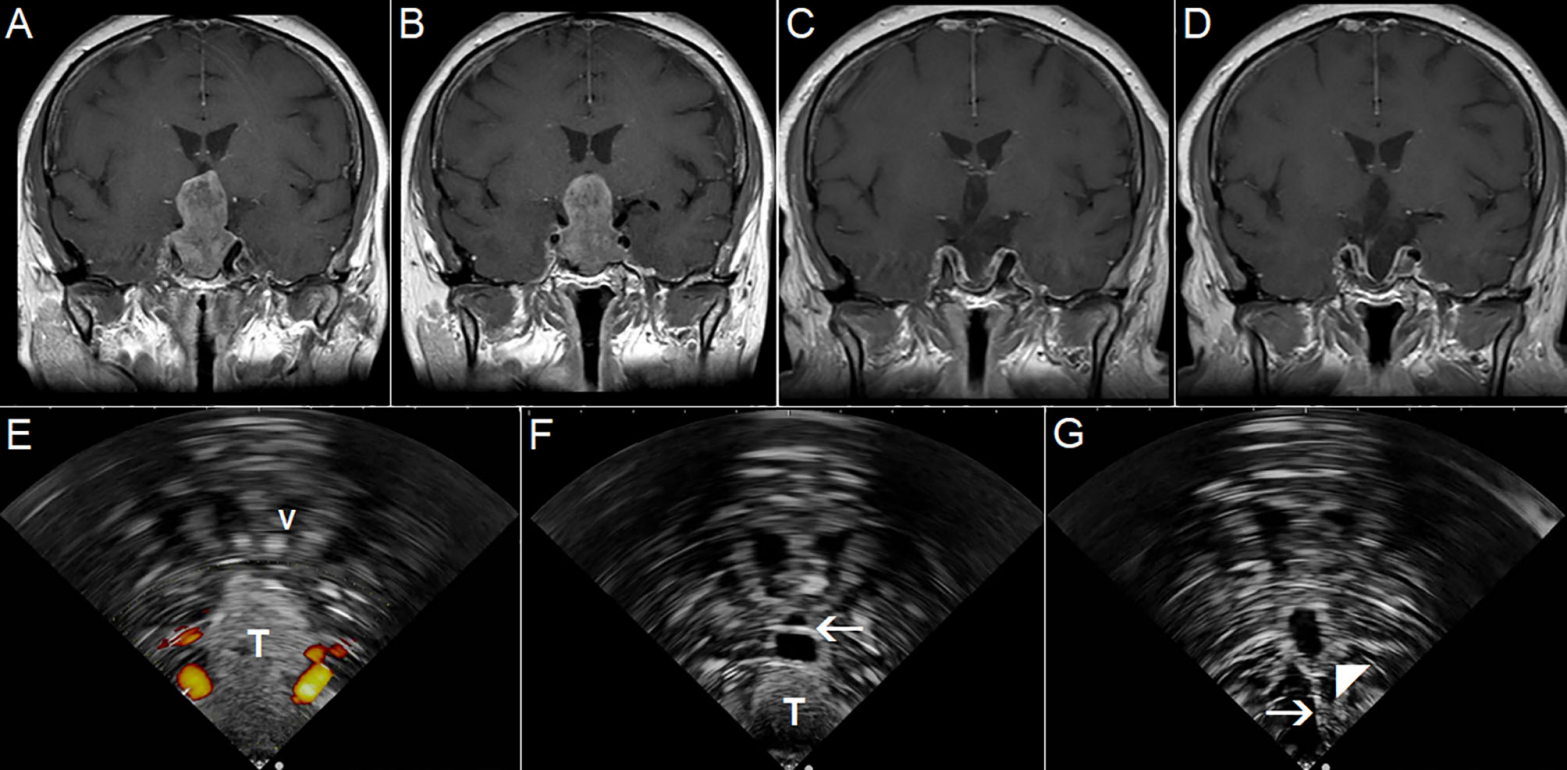
Pituitary macroadenoma invading left cavernous sinus **(A, B)** Preoperative coronal contrast-enhanced T1 MRI sequence **(C, D)** Postoperative coronal contrast-enhanced T1 MRI sequence, small tumor residuum was intentionally left in the left cavernous sinus **(E)** Pre-resectional 2D IOUS image using flexible mini-probe, note distinct depiction of the tumor tissue as well as visualization of upper segments of carotid arteries by power-Doppler mode **(F)** Intraoperative 2D IOUS image after partial tumor resection. Note partial decompression of the third ventricle with depicted interthalamic adhesion (arrow) **(G)** 2D IOUS image after tumor resection, note pituitary stalk is distinctly visible (arrow) as well as the floor of the 3rd ventricle (arrowhead). 2D, two-dimensional; MRI, magnetic resonance imaging; IOUS, intraoperative ultrasound; V, lateral ventricle; T, tumor.

Nevertheless, the available IOUS devices may still offer some important benefits during resections of pituitary tumors. Most importantly, based on the differentiation of the audio signal of a micro-Doppler probe together with neuronavigation, the position of the internal carotid artery within the adenoma-invaded cavernous sinus may be identified and an injury with brisk arterial bleeding can be avoided (73, 75). In addition, the position of the carotid artery may be identified also by its visualization using small, side-looking, high-frequency ultrasound probe (68). Next, some IOUS systems enable visualization of suprasellar space, and identification and further resection of residual suprasellar adenoma tissue (**Figure 12**) (67). This may be of particular importance during resection of giant adenomas, as the suprasellar tumor portion may be unintentionally left in place in spite of endoscope utilization. Identification of unnoticed large suprasellar residua using intraoperative imaging might be crucial to prevent hemorrhagic infarction of the tumor, compression of the hypothalamus and potentially fulminant course (76, 77). Lastly, in spite of aforementioned limitations, current IOUS use may contribute to better surgical results as compared to transsphenoidal resections without intraoperative imaging (72).

## Future Perspectives

Despite the fact that more than four decades have passed since IOUS was initially introduced during a brain tumor resection (6), this intraoperative imaging method still needs substantial improvement to achieve widespread acceptance. Nevertheless, the development of neurosurgical IOUS since the beginning of the millennium was substantial, and some pitfalls are close to reasonable solutions. On the contrary, other drawbacks need further development of the neurosurgical IOUS technology.

The prominent problem regarding ultrasound acoustic enhancement artifacts, maybe the biggest drawback of all neurosurgical IOUS devices, might soon be minimized by acoustic coupling fluid mimicking brain tissue.

Introduction of CEUS in order to guide resections of brain tumors made identification and subsequent resection of contrast enhancing malignant glioma tissue much easier. However, an important limitation is the fact that only very few commercially available ultrasound devices dedicated to neurosurgery enable CEUS, and none of them is 3D.

Interpretation of IOUS image during transsphenoidal tumor resections could be significantly easier, if new ultrasound transducers enabling distinct visualization of the sellar region in sagittal and coronal planes were developed, considering the fact that these (and not the oblique) planes are familiar to most pituitary surgeons. Development of new elongated thin ultrasound probes dedicated for intracavitary scanning might be also helpful during identification of residual glioma tissue. The important aspect of the development of new IOUS probes is the achievement of sterile intraoperative working conditions. A neurosurgical IOUS probe can be either sterilized or, if sterilization is not possible, covered with sterile sheath containing sterile coupling gel (1). Sterilization protocols of IOUS probes that contact brain tissue and cerebrospinal fluid must strictly respect regional regulations. If sterile covers are used, they should be fit tightly to the probe (1) in order to minimize the artifacts and to not alter the (special) probe shape and/or significantly enlarge the actual probe volume.

Introduction of navigated 3D IOUS reduced anatomical orientation problems caused by nonintuitive oblique planes during many types of brain surgeries by rendering IOUS images in orthogonal planes. Nevertheless, only a few studies examining effectiveness of navigated 3D IOUS utilization during transsphenoidal surgeries and CEUS-guided brain tumor resections were reported (78, 79). Research in this area should continue and result into commercially available navigated 3D IOUS systems enabling both aforementioned types of intraoperative imaging.

Correct positioning of patients before IOUS-guided resections in order to allow sufficient filling of resection cavity with fluid may be challenging; utilization of alternative adjuncts such as mini-barriers may be required. However, it is necessary to emphasize that patient positioning during intraoperative MRI-guided surgeries on the MRI table is sometimes also less-than-ideal, especially during awake tumor resections (80). Regarding awake procedures, it is worth noting that IOUS is a less time-consuming imaging modality than intraoperative MRI (50). This might be an important factor during procedures performed in conscious patients, as awake tumor resections have limited duration due to patient fatigue (81). Comparative studies examining effectiveness of both imaging modalities during awake resections should be conducted.

**Figure 13:**
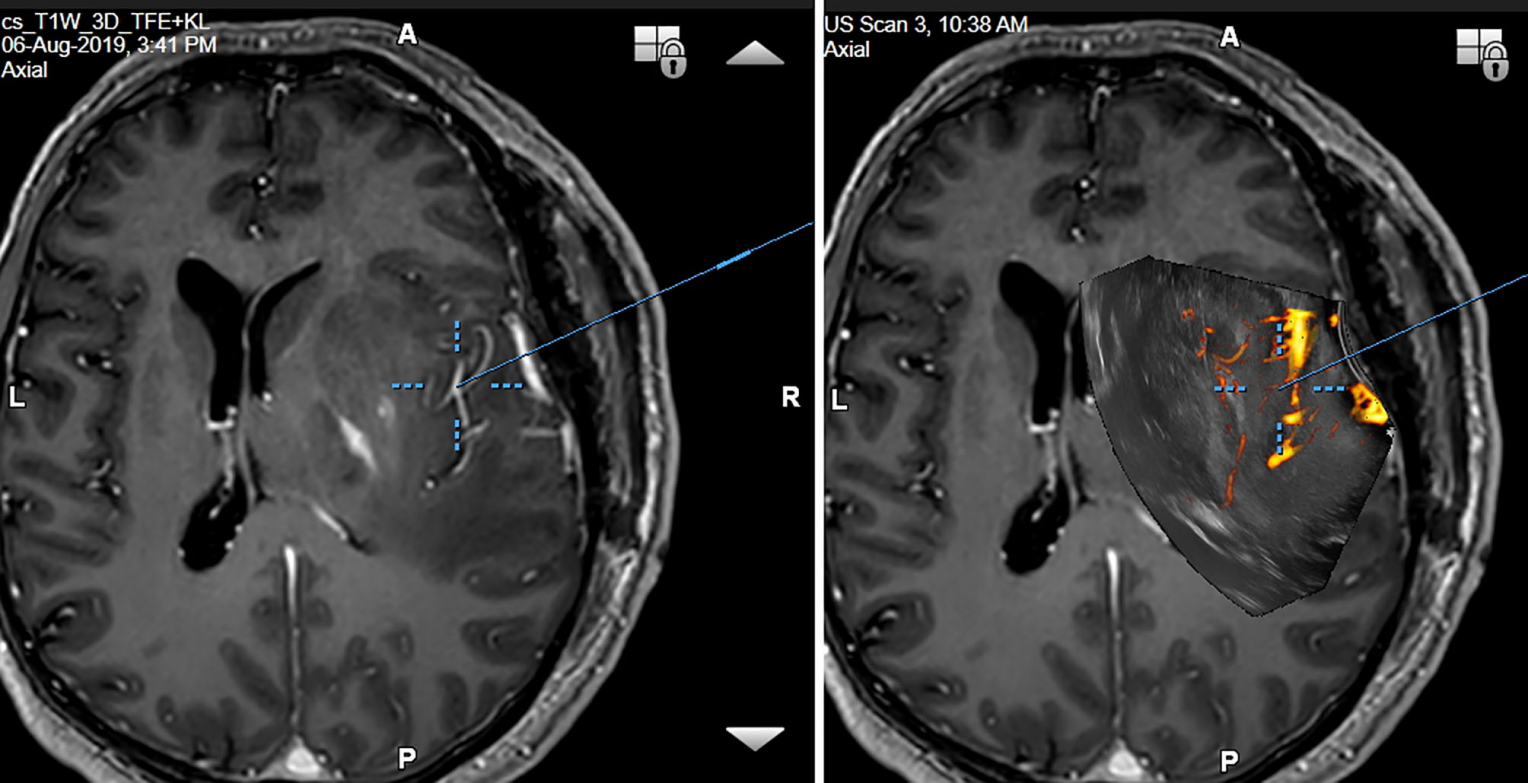
Right-sided temporo-insular glioma (reoperation after previous urgent brainstem decompression) **(Left column)** preoperative navigation 3D contrast-enhanced T1-weighted MRI sequence (3-Tesla MRI scanner) **(Right column)** pre-resectional 3D IOUS image fused with navigation MRI using a high-end navigated 3D IOUS system. Note the excellent visualization of perforating lenticulostriate arteries under the infiltrated right insula. 3D, three-dimensional; MRI, magnetic resonance imaging; IOUS, intraoperative ultrasound.

Traditional perception of intraoperative ultrasound as a modality with low image quality is slowly being overcome by innovation of ultrasound devices and ultrasound transducers. New high frequency IOUS probes may have strikingly high-resolution image. Visualization of tiny perforating arteries using power-Doppler mode seems to be at least comparable to MRI devices (**Figure 13**) (22).

Of note, as IOUS is strongly investigator-dependent, sufficient knowledge on ultrasound imaging of normal and pathological brain structures, as well as proper training are crucial for successful course of IOUS-guided resections (82). Recent IOUS simulation methods e.g. “virtual probes” (82) or IOUS-simulation smartphone applications (82), and practice on phantom (83) or animal (84) models under supervision of expert sonographers are recommended in order to refine scanning and surgical techniques (1). As showed recently by group of DiMeco, current high-end 2D IOUS systems integrated with neuronavigation may be in experienced hands of a significant benefit in terms of both extent of brain tumor resections and neurological outcomes (85). However, further prospective studies are necessary to evaluate impact of IOUS on surgical results (86, 87).

Lastly, algorithms allowing brain shift compensation based on preoperative MRI-to-IOUS rigid registration were already presented and their effectiveness was evaluated both during and after surgical procedures (88). While the rigid registration improved the alignment of the MRI and IOUS image volumes, considering the fact that brain-shift is a nonlinear process, deformable registration has the potential to further improve the results (88). Future sophisticated fusion algorithms might use IOUS image as anatomical reference, similarly to intraoperative CT, and enable deformation of preoperative 3D MRI image into “virtual intraoperative MRI” (89).

## Author Contributions

AŠ—manuscript writing, figure development, literature review, and data acquisition. JB—data acquisition and manuscript editing and review. VB—data acquisition and manuscript editing and review. AK—data acquisition and manuscript editing and review. DT—data acquisition and manuscript editing and review. AL—data acquisition and manuscript editing and review. All authors contributed to the article and approved the submitted version.

## Funding

This work was supported by the Scientific grant agency of the Ministry of education of the Slovak republic and the Slovak academy of sciences (VEGA) in the form of grant (number 1/0719/18)

## Conflict of Interest

The authors declare that the research was conducted in the absence of any commercial or financial relationships that could be construed as a potential conflict of interest.
